# Sulfites inhibit the growth of four species of beneficial gut bacteria at concentrations regarded as safe for food

**DOI:** 10.1371/journal.pone.0186629

**Published:** 2017-10-18

**Authors:** Sally V. Irwin, Peter Fisher, Emily Graham, Ashley Malek, Adriel Robidoux

**Affiliations:** University of Hawaii Maui College, Kahului, Hawaii, United States of America; Pusan National University, REPUBLIC OF KOREA

## Abstract

Sulfites and other preservatives are considered food additives to limit bacterial contamination, and are generally regarded as safe for consumption by governmental regulatory agencies at concentrations up to 5000 parts per million (ppm). Consumption of bactericidal and bacteriostatic drugs have been shown to damage beneficial bacteria in the human gut and this damage has been associated with several diseases. In the present study, bactericidal and bacteriostatic effects of two common food preservatives, sodium bisulfite and sodium sulfite, were tested on four known beneficial bacterial species common as probiotics and members of the human gut microbiota. *Lactobacillus* species *casei*, *plantarum* and *rhamnosus*, and *Streptococcus thermophilus* were grown under optimal environmental conditions to achieve early log phase at start of experiments. Bacterial cultures were challenged with sulfite concentrations ranging between 10 and 3780 ppm for six hours. To establish a control, a culture of each species was inoculated into media containing no sulfite preservative. By two hours of exposure, a substantial decrease (or no increase) of cell numbers (based on OD_600_ readings) were observed for all bacteria types, in concentrations of sulfites between 250–500 ppm, compared to cells in sulfite free media. Further testing using serial dilution and drop plates identified bactericidal effects in concentrations ranging between 1000–3780 ppm on all the *Lactobacillus* species by 4 hours of exposure and bactericidal effects on *S*. *thermophilus* in 2000ppm NaHSO_3_ after 6 hour*s* of exposure.

## Introduction

The term “sulfites” in its applications for food and drugs refers to sulfur dioxide gas; hydrogen sulfites; metabisulfites; and sulfur salts containing potassium, calcium, or sodium. These molecules are additives to beer, wine, juices, dried fruit, processed fish, seafood, meats, and some canned goods. They also occur naturally in some fermented foods as metabolites of yeast [1]. Sulfite additives are intended primarily for controlling microbial growth, preventing browning and food spoilage. Limited studies have been done to examine the effects of sulfites on lactic acid producing bacteria (LAB) prevalent during wine production which have illustrated their significant and selective inhibitory properties [2]. Under the 1958 Food Additives Amendment to the Federal Food, Drug, and Cosmetic Act, several food preservatives including sulfites were declared “Generally Regarded as Safe” (GRAS). Since that time, sulfites have been subject to multiple reviews and shown by some studies to be dangerous to humans when ingested at levels as low as 1 ppm [1,3–7]. Regulations for their use as food preservatives have changed as it has become increasingly clear that the levels of sulfites in many foods pose health concerns for some individuals. Due to insufficient statistical data regarding individual sensitivities and consumer intake levels [8,9], it has been difficult to identify the exact level at which these preservatives become harmful. Reactions can occur between these additives and primary constituents naturally present in food, as well as during preparation and digestion, contributing to this conundrum.

Currently, Health Canada lists a maximum concentration of sulfites allowable in some foods to be at 5000 ppm [10]. An acceptable daily intake (ADI) for sulfites was established by the Joint FAO/WHO Expert Committee on Food Additives (JECFA) in 1974 (0.7mg/kg body weight, expressed as sulfur dioxide, i.e. equivalent to 42 mg for a60 kg adult) and was also adopted by the Scientific Committee for Food (SCF) in 1994. The FDA requires that foods containing sulfites at concentrations greater than 10 ppm are labeled accordingly [11]. Internationally, several boards have been organized to evaluate the subject further. The Codex Committee on Food Additives (CCFA) is one such organization that has established a General Standard of Food Additives to serve as a guideline for maximum limits [8,9]. The CCFA analyzes collected data from many countries, including Germany, Australia, Brazil, Italy, France and the US. Different countries have adopted unique regulation of maximum limits of sulfites; some with national regulations much higher than suggested by the Committee. Others, despite keeping within guidelines, reported that the average consumer daily intake was much higher than their national ADI [8,11].

Analysis of sulfite concentration in red and white wine yielded a mean concentration of 70 mg/L and 122 mg/L respectively. This means that drinking about two glasses of wine (450 mL) a day equates to an intake of 75 to 130% of ADI for a 60-kg person. This statistic, combined with additional intake of sulfites common in western diet may bring the average total dietary exposure to sulfites to a total of 294% of ADI for adults and 325% for children [1,8]. It is therefore not unreasonable to consider that the average consumer is often subject to levels well over the amount generally regarded as safe. With this in mind, it is important to analyze what the impacts of excessive exposure to sulfites may have on the resident and transient microbiota of the mouth and digestive system, where they are introduced most prevalently.

The gastrointestinal tract of humans is colonized by about 800 different species of bacteria [12]. The mouth microbiome has been shown to have approximately six different phyla of bacteria with approximately 25% of the total population being *Streptococcus species* and another 12.5% made up of *Lactobacillus species* [13]. The relationship between these communities and their hosts has historically been considered commensal, though recent and ongoing studies continue to reveal benefits linked to the presence of these microflora. Research has shown these bacteria to be involved in many metabolic processes resulting in biosynthesis of molecules which act on the gut and throughout the body [12,14]. Some bacterial species produce neurotransmitters which act on the brain and affect mood. *S*. *thermophilus* generates serotonin while some *Lactobacillus* produce acetylcholine and gamma aminobutyric acid (GABA) [15]. *Lactobacillus* and *Streptococcus* species are also known to produce several B vitamins including B1 (thiamine), B2 (riboflavin), B9 (folic acid) and B12 (cobalamin). *Lactobacillus plantarum* is known to synthesize B2 in abundance. *Lactobacillus casei* is known to bind and carry thiamine, which *S*. *thermophilus* has been found to produce small amounts of, as well as pyridoxine (B6). *S*. *thermophilus* and *L*. *plantarum* synthesize folate (B9), a molecule used in cell division and construction of DNA and other genetic material [16,17]. One study suggests that lactate produced metabolically by *S*. *thermophilus* inversely impacts infection of *Clostridium difficile* in the intestinal tract of mice [18]. Noted to encourage homeostasis of the intestines, the gut microbiota also has a strong influence on development of intestinal microvilli and contributes to the strength and resilience of the overall host immune defense [12]. A connection the health of the gut microbiome and the health of the mouth microbiome has been indicated in several studies related to human diseases of the digestive and immune systems [13]. However, to date there have been no studies done to look directly at the potential effects of food preservatives on the beneficial bacteria that make up the human gut or mouth microbiome.

All four bacterial types assayed here, are commonly found in probiotic supplements as well as fermented foods. They may also be found as part of the normal gut microbiome or more often as transients that interact with the normal flora and our immune system. The *Lactobacillus* species tested were recently determined to be among the four most “robust” in their ability to survive gastric passage and to be found “metabolically active” in the ileum and colon. Additionally, ingestion of lactic acid bacteria (LAB) has been shown to positively impact the commensal gut residents by altering metabolic outputs of carbohydrate consumption while negatively affecting potential pathogens by decreasing the pH, production of biofilms that encourage the growth of commensals and the production of antimicrobials like bacteriocins and hydrogen peroxide [19].

When presented with this information, it seems pertinent to ascertain the impact that exposure to food preservatives including sulfites may have on the gut and mouth microbiota. Experiments were designed to establish a basic understanding of the effects of two types of sulfite preservatives (at concentrations deemed GRAS by the FDA) on the growth of four beneficial gut microbes.

## Methods & materials

### Bacteria cultures

Bacterial Cultures were obtained from Microbiologics in the form of LYFO DISK (lyophilized bacteria pellet). The individual cultures utilized in all experiments were as follows: *Lactobacillus plantarum* (ATCC 8014), *Lactobacillus rhamnosus* (ATCC 7469), *Lactobacillus casei* (ATCC 334) and *Streptococcus salivarius* subsp. *thermophilus* (ATCC 19258). The *Lactobacillus* species were grown and maintained on MRS agar or in MRS Broth (Difco) and *S*. *thermophilus* was grown and maintained on Brain Heart Infusion (BHI) agar or broth (Difco) at 36°C and under 5% CO_2_. All manipulations of bacteria were performed in a biological safety cabinet to limit contamination.

### Preservative assays

MRS and Brain Heart Infusion broth media were prepared according to manufacturer’s instructions within three weeks of use and kept at either 4°C (agar plates) or at room temperature (broth) before use. Anhydrous sodium sulfite (JT Baker Chemical Company) and sodium bisulfite (Arcos Organics) preservative stocks were prepared in sterile water and filter sterilized, were added to the respective media within five days of use and kept in sterile, tightly closed glass bottles to prevent out gassing of sulfur dioxide. Table 1 shows the preservatives and the concentrations tested in these assays.

**Table 1:** Sulfite preservative concentrations.

Preservative	Millimolar (mM)	Parts per million (ppm)
Na_2_SO_3_	0.1	10
Na_2_SO_3_	0.4	50
Na_2_SO_3_	0.8	100
Na_2_SO_3_	2	250
Na_2_SO_3_	4	500
Na_2_SO_3_	6	750
Na_2_SO_3_	7.9	1000
Na_2_SO_3_	16	2000
Na_2_SO_3_	30	3780
NaHSO_3_	0.1	10
NaHSO_3_	0.5	50
NaHSO_3_	1	100
NaHSO_3_	2.4	250
NaHSO_3_	4.8	500
NaHSO_3_	7.2	750
NaHSO_3_	9.6	1000
NaHSO_3_	19	2000

Sodium sulfite and sodium bisulfite concentrations in millimolar and parts per million

For each experiment, 200 milliliters (mL) of the respective broth medium was inoculated using single colonies of bacteria grown overnight on streak plates.

The Optical Density at 600nm (OD_600_) was recorded on a Perkin Elmer Victor X3 multimode plate reader after 12–15 hours of overnight (ON) growth. Culture dilutions were made to obtain an initial OD_600_ between 0.05–0.2 corresponding to approximately 1 x 10^7–8^ bacterial cells/mL for starting cultures (empirical data).

Twenty microliters (μl) of ON culture (diluted as necessary for indicated starting OD_600_) was inoculated into individual wells of 96-well plates (Spectra plate Perkin Elmer) containing180 μl of media with either no preservative added, or the indicated concentration of sulfites (Table 1). Five replicates of each test and control wells and 3 replicates of corresponding blanks were prepared. Lids of plates were coated with Triton X 100 as described by Brewster [20] to prevent condensation. Twenty-four well plates (Costar Corning cell bind surface) were prepared using 1/10 dilutions of bacteria in 1 ml of media with and without sulfite preservative and incubated for viability studies under the same conditions as the 96-well plate described above. OD_600_ readings were recorded every hour from individual replicate wells in 96-well test plates.

### Determination of bacterial growth (Cfu/mL)

**Fig 1:**
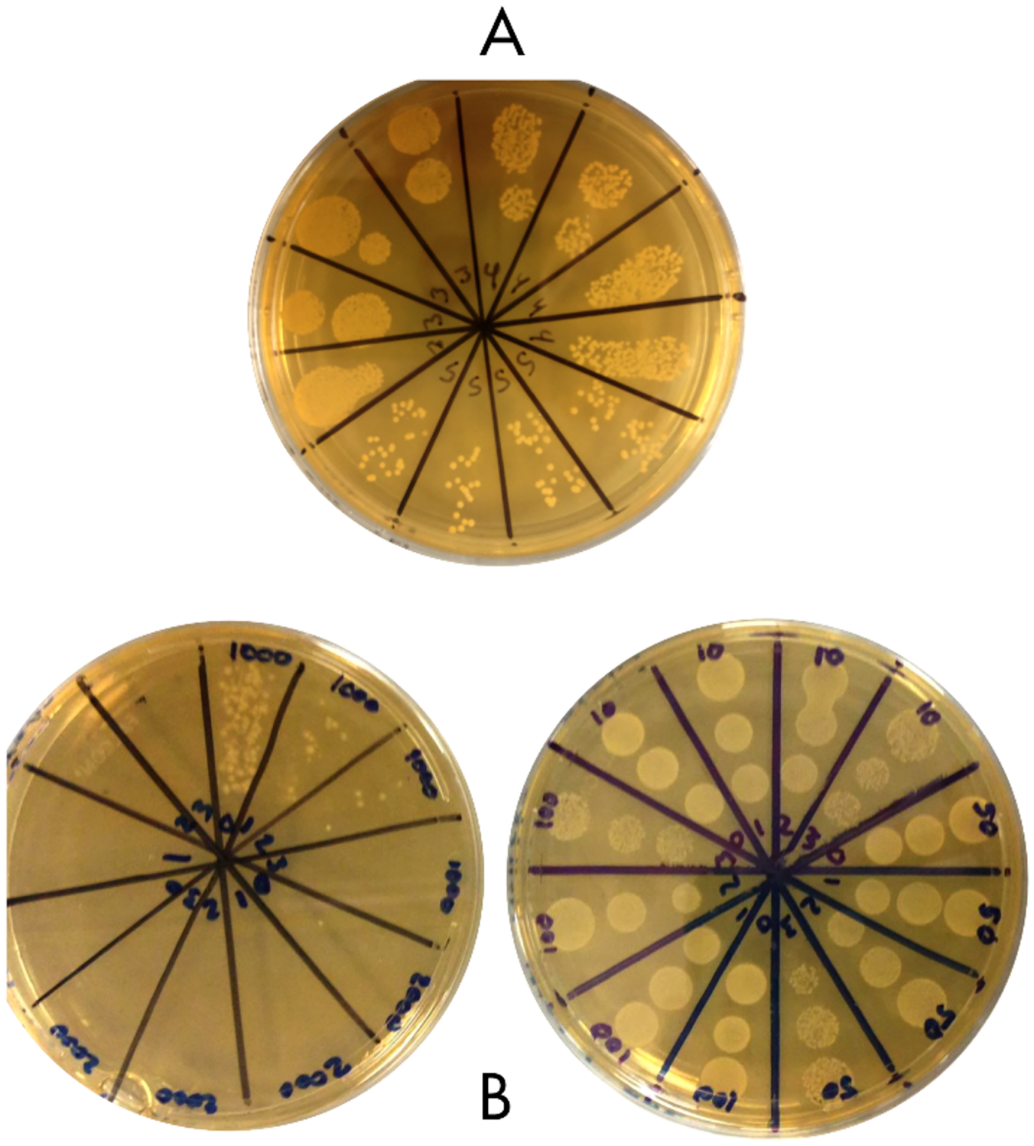
Serial dilution and drop plates. Plate “A”: Representative example of *L*. *plantarum* control (grown in sulfite free media) utilized for drop plate counts at 6 hours, in sulfite free media. Plate shows four replicates of dilutions from 10^−3^ to 10^−5^, 10μl/drop. Section labels (3, 4 and 5) correspond to dilution factors. Plate “B” Representative example of *L*. *plantarum* test plates (grown in media containing 10–50 and 1000- 2000ppm NaHSO_3_). Plate shows four replicates of dilutions from 10° to 10^−3^, 10μl/drop. Section labels (0,1,2,3) correspond to dilution factors.

Serial dilutions or undiluted samples, and drop plate counts were performed to determine the viability of bacteria between 1–6 hours of exposure time for all samples in preservative and at time 0 and 6 hours for controls. Growth studies were repeated for each bacterium a minimum of 3 times at concentrations showing inhibition of growth 1000–3780ppm, with 4 replicates for each assay. Assays for growth at concentrations between 10 and 900ppm were repeated at least twice with 4 replicates for each assay. The drop plate method [21] was modified to deliver four replicates of each diluent tested, with three samples per plate. Serial dilutions were made in a 96-well Spectra plate (Perkin Elmer) using 20 μl of each sample as described in “Preservative Assay”, into 180 μl of sterile deionized water and repeated as needed for dilutions ranging between 10^0–^10^−8^. Ten microliters of each sample were inoculated onto MRS (*Lactobacillus* species) or Brain Heart (*S*. *thermophilus*) plates that had been dried for at least 24 hours at room temperature before use. Inoculated plates were left to absorb samples for 20–30 minutes at room temperature before flipping and incubating at 36°C under 5% CO_2_ (Fig 1).

Plate counts were completed after 24 hours of incubation. Sample repeats were averaged to give an estimate of colony forming units/mL (Cfu/ml) for bacteria in wells with no preservative. Replicates of cell counts in varying concentrations of preservatives were scored as growth or no growth dependent on the appearance of bacterial colonies. In all cases, the goal was to determine the viability and growth trends of the bacteria, not the exact count of cells.

#### Statistics

**Fig 2:**
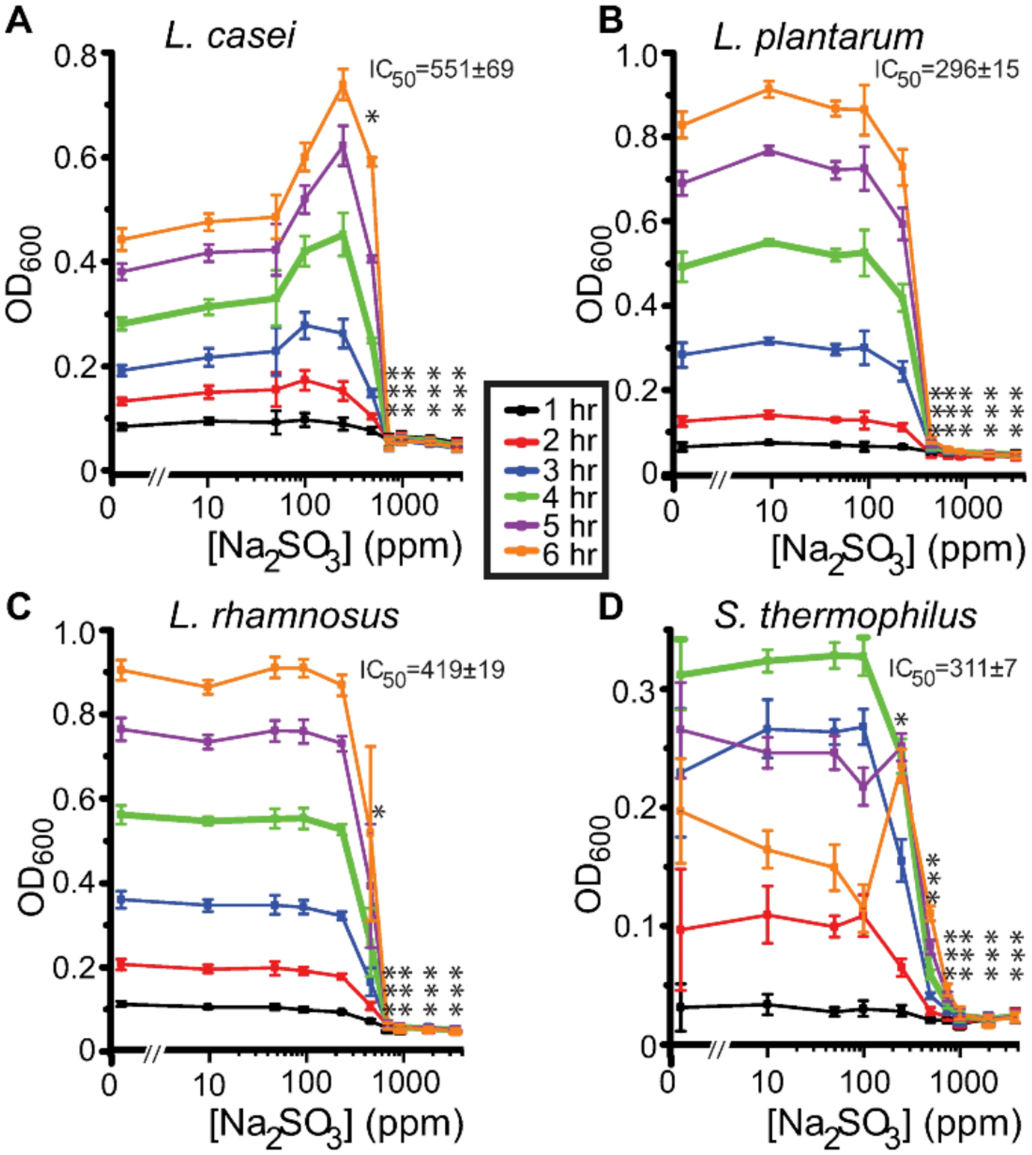
Effects of sodium sulfite on bacterial growth measured as OD_600_. Results of *Lactobacillus* species *casei*, *plantarum*, *rhamnosus* and *Streptococcus thermophilus* exposed to 9 concentrations (10–3780 ppm) of Na_2_SO_3_ over 6 hours, as compared to growth in media with no preservative (NP) is illustrated here. The IC50 of sulfites tested at 4 hours is shown for each bacterium tested. Data from uninhibited bacteria growth (dose zero) was included in the graphs however, it is not on a logarithmic scale. Significance was established by comparison of exposure to sodium sulfite vs non-exposure (*p< 0.05, **p< 0.01, ***p<0.001). Error bars indicate the standard deviation (n = 5).

**Fig 3:**
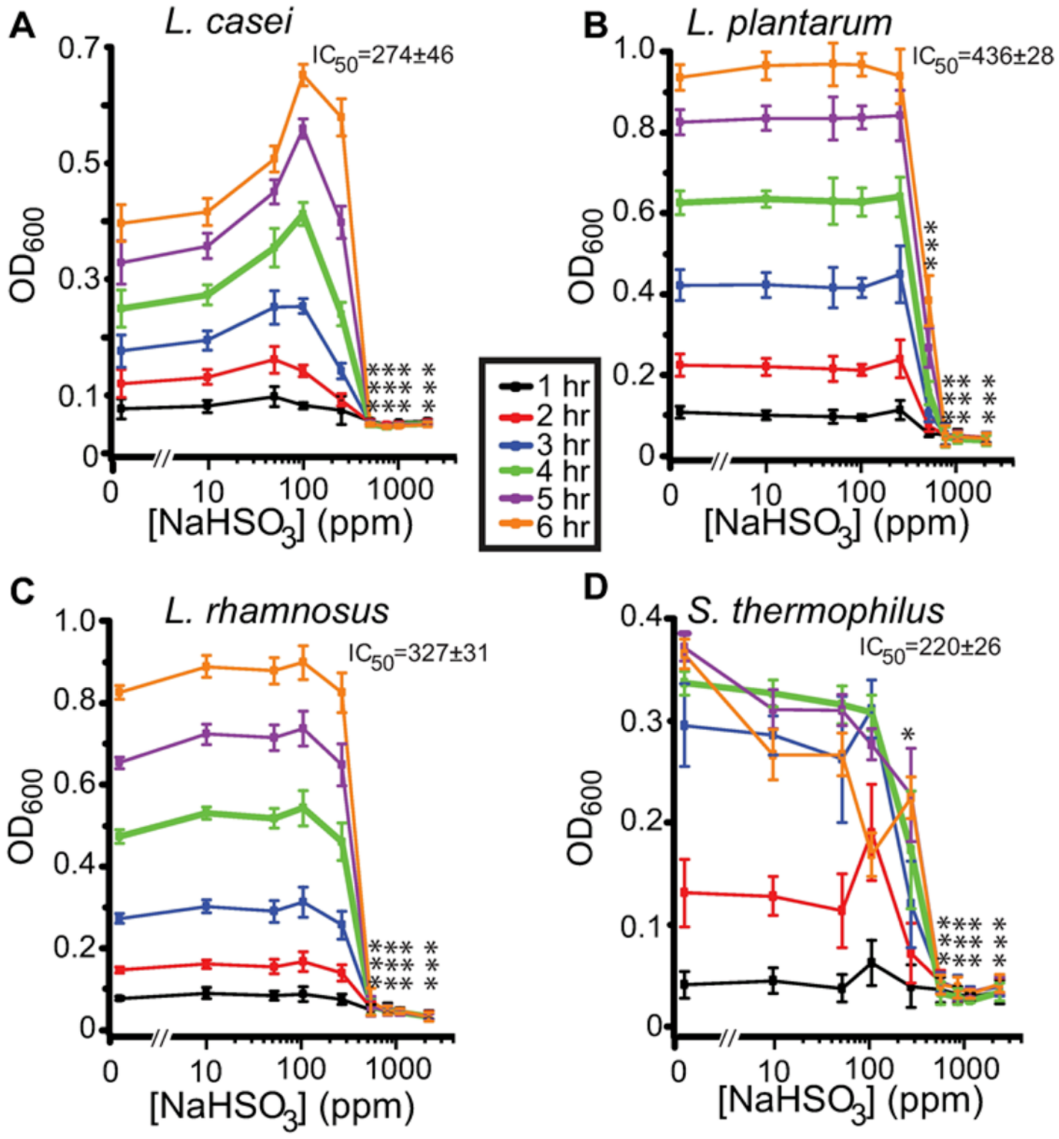
Effects of sodium bisulfite on bacterial growth measured as OD_600_. Results of *Lactobacillus* species *casei*, *plantarum*, *rhamnosus* and *Streptococcus thermophilus* exposed to 8 concentrations (10–2000 ppm) of NaHSO_3_ over 6 hours, as compared to growth in media with no preservative (NP) is illustrated here. The IC50 of sulfites tested at 4 hours is shown for each bacterium tested. Data from uninhibited bacteria growth (dose zero) was included in the graphs however, it is not on a logarithmic scale. Significance was established by comparison of exposure to sodium bisulfite vs non-exposure (*p< 0.05, **p< 0.01, ***p<0.001). Error bars indicate the standard deviation (n = 5).

For all statistical tests, a *p* value less than 0.05 was considered significant. Statistical analysis and graphic display were made with ORIGIN (Origin Lab, Northampton, MA, USA). Statistical errors were the standard deviation (SD) or standard error (SE). Average total absorbance for hours (0, 1, 2, 3, 4, 5 and 6 hours following treatment) and concentration of preservative (0, 10, 50, 100, 250, 500, 1000, 2000 and 3780 ppm) were assessed by a Two-way ANOVA, followed by post-hoc analysis with Bonferroni multi-comparison test. The IC50 for each assay at time four hours is shown in Figs 2 and 3 and for all exposure times in Table 2. This time point was chosen because the cells were in a log phase and therefore provided a consistent estimate of the inhibitory concentration.

**Table 2:** IC50 for sulfite effects on bacteria.

**NaHSO**_**3**_ **ppm**										
	***S*. *thermophilus***			***L*. *plantarum***			***L*. *casei***		***L*. *rhamnosus***	
**Hour**	**IC50**	**Error +/-**		**IC50**	**Error +/-**		**IC50**	**Error +/-**	**IC50**	**Error +/-**
**2**	241.68	59.34		448.17	1578.84		244.81	16.16	340.03	31.96
**3**	223.12	21.75		439.85	199.49		253.57	18.57	328.24	27.42
**4**	219.56	25.74		435.89	27.96		274.09	45.83	326.88	30.88
**5**	169.59	36.68		458.47	12.69		295.22	68.95	329.06	32.67
**6**	121.36	63.62		477.92	11.91		###	###	342.04	24.21
**Na**_**2**_**SO**_**3**_ **ppm**										
	***S*. *thermophilus***			***L*. *plantarum***			***L*. *casei***		***L*. *rhamnosus***	
**Hour**	**IC50**		**Error +/-**	**IC50**		**Error +/-**	**IC50**	**Error +/-**	**IC50**	**Error +/-**
**2**	262.81		13.94	571.79		22.98	522.83	34.64	432.80	17.94
**3**	261.86		10.56	556.87		17.65	528.42	38.36	405.03	20.52
**4**	311.98		6.99	551.70		17.63	551.37	69.03	419.36	19.46
**5**	416.26		23.88	556.32		17.71	585.13	116.08	493.89	29.96
**6**	533.19		46.26	567.09		16.03	###	###	508.36	39.01

Table displays concentration of sulfites that was calculated at 50% inhibition of bacterial growth based on OD_600_ (Figs 2 and 3). IC50 data for *L*. *casei* at 6 hours (###) was uncertain.

## Results

### Preservative assays

#### Relative cell number based on OD_600_

Within 2 hours of sulfite exposure, all bacteria types showed either a decrease or no increase in cell numbers in concentrations of preservatives between 250–750 ppm based on OD_600_ readings. This indicated a bacteriostatic effect from both types of sulfites tested on all bacteria. A steady increase in cell numbers was observed in all samples not containing preservative (NP) over the six hour exposure time (Figs 2 and 3), except for *S*.*thermophilus* which saw a slight decrease in cell numbers as indicated by OD_600_ readings, between hours five and six when grown in the 96-well plate. This decrease in growth for *S*. *thermophilus* was not observed in replicate experiments (Table’s C and E in S1 Table) when bacteria were grown in 24-well plates. It is hypothesized that this decrease was due to an exhaustion of nutrients in the 96-well plate. This is supported by the drop plate data from the same experiments which showed continued growth of bacteria at 6 hours. An increase in growth was observed by *L*. *casei* in low concentrations of both sulfites ranging from 50–250ppm. The sulfites at these concentrations seem to be acting as a nutrient.

#### Viability based on cell growth

Serial dilutions and plate counts to look for cell growth were performed to test the viability of the cells throughout exposure to preservatives. Plate counts were initiated after 24 hours of incubation time and revealed a difference between *Lactobacillus* species and *Streptococcus thermophilus* in relation to the bactericidal effects of the sulfites tested. Plates were observed for an additional 24 hours to look for any late growth. Some random, low level growth (less than 10 cells/ml) after 48 hours incubation was observed 2–3 times throughout all experiments, but it was inconsistent in relation to preservative being tested or incubation time. We hypothesize that these late growing colonies may be resistant to the sulfites. Table 3 summarizes the results from these tests and supporting data can be found in supplemental tables A,B, D and F in S1 Table.

**Table 3:** Bactericidal effects of sulfites using serial dilutions and drop plates.

Sulfite Tested	Bacteria type	Dose and exposure time of bactericidal effects	Range of CFU’s/ml of bacteria not treated with sulfite (6 hours)
NaHSO_3_	*L*. *plantarum*	≥ 4 hours ≥ 1000 ppm	3.50E8–5.4E9
	*L*. *casei*	≥ 2 hours ≥ 1000 ppm	3.80E7–4.6E9
	*L*. *rhamnosus*	≥ 2 hours ≥ 1000 ppm	7.25E7—3E8
	*S*.*thermophilus*	≥ 6 hours ≥ 2000 ppm	3.90E9—1E10
Na_2_SO_3_	*L*. *plantarum*	≥4 hours ≥ 3780 ppm ≥6 hours ≥ 2000 ppm	3.50E8–5.10E9
	*L*. *casei*	≥ 4 hours ≥ 3780 ppm ≥ 5 hours ≥ 1000 ppm	2.8E8–3.80E7
	*L*. *rhamnosus*	≥ 4 hours ≥ 1000 ppm	2.20E7–2.6E8
	*S*. *thermophilus*	No bactericidal dose observed between 1–6 hours.	3.90E9–1.0E10

Bactericidal effects after exposure to sulfites between 1000–3780 ppm were recorded from two separate experiments, (each with four replicates). Results shown have a confidence level of 100% based on the observation of no growth (lack of colonies) in all replicates (n = 8) as compared to growth (presence of colonies) with the same bacteria not exposed to sulfites (control). The range of colony forming units (CFU’s)/ milliliter of untreated bacteria at 6 hours exposure time from two experiments establishes a baseline for uninhibited (no sulfite exposure) growth.

#### Sodium sulfite

All three *Lactobacillus* species stopped increasing in number within 2 hrs of exposure at concentrations between 250–750 ppm NaSO_3_, (Fig 2), and were found to be non-viable within 4 hours of exposure, at concentrations ranging between 1000 -3780ppm, dependent on species tested (Table 3). *Streptococcus thermophilus* also stopped increasing in number within two hours of exposure at concentrations between 250–500ppm; however, the bacteria were still viable in all concentrations of sodium sulfite tested up to 6 hours exposure time. These results indicate a bacteriostatic effect from sodium sulfite on all bacteria within two hours of exposure and a bactericidal effect on all the *Lactobacillus* species by 4 hours of exposure.

#### Sodium bisulfite

All bacteria stopped increasing in cell number within two hours of exposure at sulfite concentrations between 250–500 ppm NaHSO_3_, (Fig 3). Sodium bisulfite was observed to be bactericidal at 2 hours exposure to *L*. *casei* and *L*. *rhamnosus* at 1000ppm. *Lactobacillus plantarum* was found to be non-viable by 4 hours exposure at ≥ 1000ppm. Sodium bisulfite was bactericidal to *S*. *thermophilus* at 6 hours exposure and ≥ 1000 ppm. Results shown in Table 3.

## Discussion

In the most recent evaluation of sulfite consumption by the World Health Organization, it was concluded that in many countries, a significant portion of the population regularly exceeds the daily amounts of sulfites considered “‘safe” in their diets [8]. In addition to food, exposure to sulfites may occur through the use of cosmetics such as hair dyes and bleaches, perfumes and creams as well as some topical or parenteral medications. When food preservatives were widely introduced in the early 1960’s, testing was done to determine the effectiveness of the additives to prevent pathogen growth in food and potential damage to host cells. However, the possible effects on beneficial bacteria were not evaluated.

The molecular mechanisms of sulfite antimicrobial actions have not been clearly defined. However, several plausible reactions have been proposed that are useful for a general understanding in biological systems where the oxidation of sulfite and the subsequent chain reactions provides reactive intermediates capable of adversely reacting with biological molecules [22,23]. Sulfite is a strong nucleophile and reacts with many biomolecules by substitution at electrophilic positions causing a myriad of potential cell damaging reactions. These reactive molecules have been shown to inhibit key enzymes in living cells such as those involved in ATP and NADH production leading to cell death.

Facultative heterofermentative lactic acid producing bacteria (LAB), *Lactobacillus casei*, *L*. *rhamnosus and L*. *plantarum* showed similar and higher susceptibility to sulfites than the homofermentative LAB *Streptococcus thermophilus*. One possible difference in their susceptibilities may be the formation of the highly toxic sulfur trioxide anion radicals (STAR), which are known to be formed by the reaction of sulfite with H_2_O_2_ [22–24]. All the bacteria tested in this study are known to produce variable amounts of hydrogen peroxide (H_2_O_2_) dependent on environmental conditions [25]. It would be reasonable to suggest one of the factors contributing to cell death by sulfites of LAB would be the available amount of H_2_O_2_. However, it has been observed that the level of H_2_O_2_ produced is inversely related to the amount of glucose present, and relative to specific pH, nitrogen source, temperature and duration of growth, making it difficult to predict [26]. We suggest that another possible difference in sulfite susceptibility of *Streptococcus thermophilus* compared to *Lactobacillus spp* may be that *Streptococcus thermophilus* produces less acid in BHI media due to the lower glucose content as compared to growth in MRS media, (MRS has 10 times more glucose than BHI) and therefore less of the damaging sulfur dioxide would be generated.

Sulfite is oxidized to sulfate in mammals by the mitochondrial enzyme sulfite oxidase, found predominantly in liver and kidney cells [22]. The concentration of sulfites an individual can metabolize daily is variable based on the amount of the enzyme being produced and individual genetic and environmental conditions. There are several human disease conditions or symptoms connected to sulfite exposure. For some people sulfite exposure induces signs and symptoms associated with allergic responses and other symptoms, such as dermatitis/urticarial, flushing, hypotension, abdominal pain and diarrhea to life-threatening anaphylactic and asthmatic reactions [3]. The rapid death of the bacteria observed in the presence of sulfites could release toxic and allergenic compounds such as lipopolysaccharides or peptidoglycan from lysed bacteria. The released substances may be one of the mechanisms for the various damaging biological activities observed in human sulfite reactions [4, 6, and 7].

The production of several essential B vitamins is attributed to members of the gut microbiota and transients found in fermented foods [17–18]. Thiamine deficiency has been observed in patients with obesity, diabetics and alcoholics [27]. It has been established that sulfites break down thiamine (B1) and foods containing thiamine are restricted for use of sulfite preservatives for this reason [8]. Sulfites found in some alcohol and processed foods may contribute to thiamine deficiency due to the breakdown of thiamine present as well as limit the production of thiamine by certain probiotic or commensal bacteria susceptible to sulfites. Other studies have shown a direct correlation between obesity and chronic inflammation due to different pathogenic mechanisms [28]. Obese individuals have been shown to have an increase in the number of gram negative bacteria in their guts leading to an increase in lipopolysaccharides (LPS) from the dying cells. Interestingly, studies have shown an increase in leptin inhibition in lipopolysaccharide (LPS) treated murine adipocytes exposed to sodium sulfites [29,30]. This subsequently causes a decrease in satiety leading to a possible link with sulfites and their effects on gut bacteria to the increase in obesity and related illnesses.

The bacteria utilized for the described experiments are commonly used as probiotics and are found in fermented foods. Studies have shown health benefits of consuming probiotics for several different conditions including but not limited to diarrhea [31], during or following antibiotic treatment [31–33], to support proper function of the nervous system [34] and weight loss [35]. Several studies on the human gut inhabitants have revealed that antibiotic use [36,37], most notably in infants, and to a lesser extent in adults, disrupt normal flora. *Clostridium difficile* overgrowth is often initiated in response to prolonged antibiotic use and is one of many pathogens as well as some commensal organisms that can reduce sulfites and therefore are not susceptible to these preservatives [38,39]. This suggests that similar to antibiotics, the use of sulfites in food may promote the growth of these organisms while limiting the growth of many beneficial, sulfite sensitive bacteria. Ironically, *Streptococcus thermophilus* has been shown to reduce the growth of *C*. *difficile* due to production of lactate [38], however as we have observed, it is inhibited by sulfites. Diminishment of beneficial microbes and their metabolites by sulfites in the human gut and mouth may contribute to an increase in the probability of higher populations of pathogens and thus adversely affect human health.

## Conclusions

Approximately 80% of the digestive process takes place in the small and large intestines where most of the gut microbiota resides. Depending on the composition of meals, food spends 2–6 hours being absorbed in the small intestine and 10–59 hours in the colon before elimination as feces [40–41]. The experiments described here exposed four beneficial bacterial species to varying concentrations of sulfites for 6 hours. This is a much shorter time frame to assay the effects of the preservative than actual exposure time would be in the digestive system. The bacteria were challenged with the preservatives under their optimal growth conditions, making this a stringent test. Despite the short exposure time and optimal environmental growth conditions, all four bacteria exhibited either bactericidal or bacteriostatic effects from sulfites in concentrations that fell well within the ADI range determined by the FDA and other similar regulatory bodies [8,10,11].

Through these experiments we observed a substantial impact on the viability of the bacteria tested under in-vitro conditions. While the results were noteworthy, it is obvious to us that the conditions in the human digestive system, such as a dramatically lower pH in the stomach, may very well change these effects by altering the sulfites, making the bacteria more or less susceptible to the observed inhibition. The bacteriostatic and bactericidal effects suggest that these preservatives may be altering the gut and/or the mouth microbiome. Therefore, it would be worth further examination as a possible contributor to diseases related to a dysbiotic human microbiota. Future work will be to test sulfites at longer exposure times and in both artificial digestion conditions and ultimately in-vivo using mouse or human studies.

## Supporting information

S1 Table Viability and growth data described in manuscript. (DOCX)
