# Supplementary material for: Sulfites inhibit the growth of four species of beneficial gut bacteria at concentrations regarded as safe for food
Source: PLoS One. 2017 Oct 18;12(10):e0186629. doi: 10.1371/journal.pone.0186629 (PMC5646858; doi:10.1371/journal.pone.0186629)
Supplement: S1 Table — (DOCX) [file pone.0186629.s001.docx]

**Table S1: Viability and Growth Data Described in Manuscript**

**Table S1A: Growth of Bacterial Colonies After Exposure to Sodium Sulfite [Na₂SO₃]**

| *L. casei* | | | | | | | | *L. plantarum* | | | | | | | |
| --- | --- | --- | --- | --- | --- | --- | --- | --- | --- | --- | --- | --- | --- | --- | --- |
|  | 0hr | 1hr | 2hr | 3hr | 4hr | 5hr | 6hr |  | 0hr | 1hr | 2hr | 3hr | 4hr | 5hr | 6hr |
| 10ppm |  | 1 | 1 |  | 1 | 1 | 1 | 10ppm |  | 1 | 1 | 1 | 1 | 1 | 1 |
| 50ppm |  | 1 | 1 |  | 1 | 1 | 1 | 50ppm |  | 1 | 1 | 1 | 1 | 1 | 1 |
| 100ppm |  | 1 | 1 |  | 1 | 1 | 1 | 100ppm |  | 1 | 1 | 1 | 1 | 1 | 1 |
| 250ppm |  | 1 | 1 | 1 | 1 | 1 | 1 | 250ppm |  | 1 | 1 | 1 | 1 | 1 | 1 |
| 500ppm |  | 1 | 1 | 1 | 1 | 1 | 1 | 500ppm |  | 1 | 1 | 1 | 1 | 1 | 1 |
| 750ppm |  | 1 | 1 | 0 | 0 | 1 | 1 | 750ppm |  | 1 | 1 | 1 | 1 | 1 | 1 |
| 1000ppm |  | 1 | 0 | 0 | 0 | 0 | 0 | 1000ppm |  | 1 | 1 | 1 | 1 | 1 | 1 |
| 2000ppm |  | 1 | 0 | 0 | 0 | 0 | 0 | 2000ppm |  | 1 | 1 | 1 | 1 | 1 | 0 |
| 3780ppm |  | 1 | 0 | 0 | 0 | 0 | 0 | 3780ppm |  | 1 | 1 | 1 | 0 | 0 | 0 |
| NP | 7.9E6 |  |  |  |  |  | 8.8E7 | NP | 1.8E6 |  |  |  |  |  | 3.5E8 |
| *L. rhamnosus* | | | | | | | | *S. thermophilus* | | | | | | | |
|  | 0hr | 1hr | 2hr | 3hr | 4hr | 5hr | 6hr |  | 0hr | 1hr | 2hr | 3hr | 4hr | 5hr | 6hr |
| 10ppm |  | 1 | 1 |  | 1 | 1 | 1 | 10ppm |  | 1 | 1 | 1 | 1 | 1 | 1 |
| 50ppm |  | 1 | 1 |  | 1 | 1 | 1 | 50ppm |  | 1 | 1 | 1 | 1 | 1 | 1 |
| 100ppm |  | 1 | 1 |  | 1 | 1 | 1 | 100ppm |  | 1 | 1 | 1 | 1 | 1 | 1 |
| 250ppm |  | 1 | 1 |  | 1 | 1 | 1 | 250ppm |  | 1 | 1 | 1 | 1 | 1 | 1 |
| 500ppm |  | 1 | 1 |  | 1 | 1 | 1 | 500ppm |  | 1 | 1 | 1 | 1 | 1 | 1 |
| 750ppm |  | 1 | 1 |  | 1 | 1 | 1 | 750ppm |  | 1 | 1 | 1 | 1 | 1 | 1 |
| 1000ppm |  | 1 | 1 |  | 0 | 0 | 0 | 1000ppm |  | 1 | 1 | 1 | 1 | 1 | 1 |
| 2000ppm |  | 1 | 1 |  | 0 | 0 | 0 | 2000ppm |  | 1 | 1 | 1 | 1 | 1 | 1 |
| 3780ppm |  | 0 | 0 |  | 0 | 0 | 0 | 3780ppm |  | 1 | 1 | 1 | 1 | 1 | 1 |
| NP | 4.6E6 |  |  |  |  |  | 2.2E7 | NP | 1.3E8 |  |  |  |  |  |  |

Table S1A: Data set describing CFU/ml readings in Na_2_SO_3_. One “1” represents appearance of bacterial colonies (growth), zeros “0” represent no growth (no colonies observed) 24 hours after inoculation. Each experiment had 4 replicates/concentration of preservative tested. Data used to determine the results of bactericidal studies summarized in Table 3 in manuscript (along with data from Table S4)

**Table S1B: Growth of Bacterial Colonies After Exposure to Sodium Bisulfite [NaHSO₃]**

| *L. casei* | | | | | | | | *L. plantarum* | | | | | | | |
| --- | --- | --- | --- | --- | --- | --- | --- | --- | --- | --- | --- | --- | --- | --- | --- |
|  | 0hr | 1hr | 2hr | 3hr | 4hr | 5hr | 6hr |  | 0hr | 1hr | 2hr | 3hr | 4hr | 5hr | 6hr |
| 10ppm |  | 2 | 1 | 1 | 1 | 1 | 1 | 10ppm |  | 1 | 1 | 1 | 1 | 1 | 1 |
| 50ppm |  | 1 | 1 | 1 | 1 | 1 | 1 | 50ppm |  | 1 | 1 | 1 | 1 | 1 | 1 |
| 100ppm |  | 1 | 1 | 1 | 1 | 1 | 1 | 100ppm |  | 1 | 1 | 1 | 1 | 1 | 1 |
| 250ppm |  | 1 | 1 | 1 | 1 | 1 | 1 | 250ppm |  | 1 | 1 | 1 | 1 | 1 | 1 |
| 500ppm |  | 1 | 1 | 0 | 0 | 0 | 0 | 500ppm |  | 1 | 1 | 1 | 1 | 1 | 1 |
| 750ppm |  | 1 | 1 | 0 | 0 | 0 | 0 | 750ppm |  | 1 | 1 | 1 | 1 | 1 | 1 |
| 1000ppm |  | 1 | 0 | 0 | 0 | 0 | 0 | 1000ppm |  | 1 | 1 | 1 | 0 | 0 | 0 |
| 2000ppm |  | 1 | 0 | 0 | 0 | 0 | 0 | 2000ppm |  | 1 | 1 | 0 | 0 | 0 | 0 |
| NP | 4.4E6 |  |  |  |  |  | 3.8E7 | NP | 3.3E7 |  |  |  |  |  | 3.5E8 |
| *L. rhamnosus* | | | | | | | | *S. thermophilus* | | | | | | | |
|  | 0hr | 1hr | 2hr | 3hr | 4hr | 5hr | 6hr |  | 0hr | 1hr | 2hr | 3hr | 4hr | 5hr | 6hr |
| 10ppm |  |  |  |  | 1 | 1 | ND | 10ppm |  | 1 | 1 |  | 1 | 1 | 1 |
| 50ppm |  | 1 | 1 | 1 | 1 | 1 | 1 | 50ppm |  | 1 | 1 | 1 | 1 | 1 | 1 |
| 100ppm |  | 1 | 1 | 1 | 1 | 1 | 1 | 100ppm |  | 1 | 1 | 1 | 1 | 1 | 1 |
| 250ppm |  | 1 | 1 | 1 | 1 | 1 | 1 | 250ppm |  | 1 | 1 | 1 | 1 | 1 | 1 |
| 500ppm |  | 1 | 1 | 1 | 1 | 1 | 1 | 500ppm |  | 1 | 1 | 1 | 1 | 1 | 1 |
| 750ppm |  | 1 | 0 | 0 | 0 | 0 | 0 | 750ppm |  | 1 | 1 | 1 | 1 | 1 | 1 |
| 1000ppm |  | 0 | 0 | 0 | 0 | 0 | 0 | 1000ppm |  | 1 | 1 | 1 | 1 | 1 | 0 |
| 2000ppm |  | 0 | 0 | 0 | 0 | 0 | 0 | 2000ppm |  | 1 | 1 | 1 | 1 | 1 | 0 |
| NP | 4.7E6 |  |  |  |  |  | 7.25E7 | NP | 1.3E8 |  |  |  |  |  | 3.9E9 |

Table S1B: Data set describing CFU/ml readings in NaHSO_3_. One “1” represents appearance of colonies, zeros “0” represent no growth (no colonies observed) 24 hours after inoculation. Each experiment had 4 replicates /concentration of preservative tested. Data used to determine the results of bactericidal studies summarized in Table 3 in manuscript (along with data from Table S5)

**Table S1C: OD600 Readings for Sodium Sulfite (Na₂SO₃) Assays – Experiments done with single readings on Thermo Fisher UV-VIS Spectrophotometer**

| OD600 L. casei in Sodium Sulfite 11/17/2016 | | | | | | OD600 L. plantarum in Sodium Sulfite 10/04/2016 | | | | | |
| --- | --- | --- | --- | --- | --- | --- | --- | --- | --- | --- | --- |
| Time/  Hours | NP | 1260  ppm | 1890  ppm | 2520  ppm | 3780  ppm | Time/  Hours | NP | 1260  ppm | 1890  ppm | 2520  ppm | 3780  ppm |
| 0 | 0.052 | 0.052 | 0.052 | 0.052 | 0.052 | 0 | 0.150 | 0.150 | 0.150 | 0.150 | 0.150 |
| 1 | 0.078 | 0.066 | 0.051 | 0.047 | 0.047 | 1 | 0.381 | 0.224 | 0.219 | 0.206 | 0.204 |
| 2 | 0.081 | 0.056 | 0.047 | 0.042 | 0.050 | 2 | 0.575 | 0.208 | 0.207 | 0.189 | 0.181 |
| 3 | 0.124 | 0.051 | 0.045 | 0.043 | 0.040 | 3 | 1.043 | 0.175 | 0.201 | 0.196 | 0.179 |
| 4 | 0.190 | 0.043 | 0.045 | 0.047 | 0.042 | 4 | 1.385 | 0.178 | 0.199 | 0.171 | 0.180 |
| 5 | 0.272 | 0.056 | 0.048 | 0.045 | 0.050 | 5 | 1.602 | 0.241 | 0.221 | 0.247 | 0.229 |
| 6 | 0.359 | 0.056 | 0.034 | 0.010 | 0.034 | 6 | 1.740 | 0.227 | 0.241 | 0.249 | 0.179 |
| OD600 L. rhamnosus in Sodium Sulfite 12/14/2016 | | | | | | OD600 S. thermophilus in Sodium Sulfite 10/27/2016 | | | | | |
| Time/  Hours | NP | 1260  ppm | 1890  ppm | 2520  ppm | 3780  ppm | Time/  Hours | NP | 1260  ppm | 1890  ppm | 2520  ppm | 3780  ppm |
| 0 | 0.107 | 0.107 | 0.107 | 0.107 | 0.107 | 0 | 0.044 | 0.044 | 0.044 | 0.044 | 0.044 |
| 1 | 0.141 | 0.103 | 0.101 | 0.108 | 0.108 | 1 | 0.081 | 0.051 | 0.057 | 0.048 | 0.046 |
| 2 | 0.249 | 0.187 | 0.170 | 0.163 | 0.174 | 2 | 0.149 | 0.056 | 0.049 | 0.045 | 0.048 |
| 3 | 0.428 | 0.097 | 0.099 | 0.104 | 0.102 | 3 | 0.405 | 0.049 | 0.054 | 0.042 | 0.047 |
| 4 | 0.689 | 0.102 | 0.100 | 0.104 | 0.105 | 4 | 0.584 | 0.053 | 0.050 | 0.046 | 0.049 |
| 5 | 0.857 | 0.103 | 0.102 | 0.106 | 0.101 | 5 | 0.821 | 0.097 | 0.074 | 0.069 | 0.050 |
| 6 | 1.115 | 0.093 | 0.101 | 0.099 | 0.100 | 6 | 0.893 | 0.087 | 0.064 | 0.044 | 0.049 |

Table S1C: Data set describing OD600 data for bacteria in Na_2_SO_3_.

**Table S1D: Colony Forming Units After Exposure to Sodium Sulfite (Na₂SO₃)**

| CFU/mL L. casei in Sodium Sulfite 11/17/2016 | | | | | | CFU/mL L. plantarum in Sodium Sulfite 10/04/2016 | | | | | |
| --- | --- | --- | --- | --- | --- | --- | --- | --- | --- | --- | --- |
| Time/  Hours | NP | 1260  ppm | 1890  ppm | 2520  ppm | 3780  ppm | Time/  Hours | NP | 1260  ppm | 1890  ppm | 2520  ppm | 3780  ppm |
| 0 | 5.2E7 | 5.2E7 | 5.2E7 | 5.2E7 | 5.2E7 | 0 | 4.85E7 | 4.85E7 | 4.85E7 | 4.85E7 | 4.85E7 |
| 2 |  | 5.4E5 | 0 | 0 | 0 | 2 |  | 1.60E3 | 1.25E2 | 2.60E2 | 3.75E2 |
| 4 |  | 0 | 0 | 0 | 0 | 4 |  | 0 | 0 | 0 | 0 |
| 6 | 2.8E8 | 0 | 0 | 0 | 0 | 6 | 5.10E9 | 0 | 0 | 0 | 0 |
| CFU/mL L. rhamnosus is Sodium Sulfite 12/14/2016 | | | | | | CFU/mL S. thermophilus in Sodium Sulfite 10/27/2016 | | | | | |
| Time/  Hours | NP | 1260  ppm | 1890  ppm | 2520  ppm | 3780  ppm | Time/  Hours | NP | 1260  ppm | 1890  ppm | 2520  ppm | 3780  ppm |
| 0 | 9.5E6 | 9.5E6 | 9.5E6 | 9.5E6 | 9.5E6 | 0 | 8.25E7 | 8.25E7 | 8.25E7 | 8.25E7 | 8.25E7 |
| 2 |  | 2.0E2 | 4.5E3 | 2.0E2 | 2.0E2 | 2 |  | 2.0E7 | 2.1E7 | 2.0E7 | 3.2E7 |
| 4 |  | 0 | 0 | 0 | 0 | 4 |  | 4.37E7 | 1.5E5 | 1.5E5 | 1.5E5 |
| 6 | 2.6E8 | 0 | 0 | 0 | 0 | 6 | 1.0E10 | 1E5^a^ | 1E5^a^ | 1E5^a^ | 1E5^a^ |

Table S1D: Data set describing CFU/ml readings in Na_2_SO_3_. Results are estimated based on 3-8 replicates from drop plates. Numbers with superscript “a” are based on estimates at dilutions of 10^-2^ replicates that had too many colonies to accurately count. Zeros “0” represent no growth observed after 24 hours of inoculation. Data used to determine the results of bactericidal studies summarized in Table 3 in manuscript (along with data from Table S1F)

**Table S1E: OD600 Readings for Sodium Bisulfite (NaHSO₃) Assays- Experiments done with single readings on Thermo Fisher UV-VIS Spectrophotometer**

| OD600 L. casei in Sodium Bisulfite 1/31/2017 | | | | | | OD600 L. plantarum in Sodium Bisulfite 1/31/2017 | | | | | |
| --- | --- | --- | --- | --- | --- | --- | --- | --- | --- | --- | --- |
| Time/  Hours | NP | 900  ppm | 1170  ppm | 1350  ppm | 1800  ppm | Time/  Hours | NP | 900  ppm | 1170  ppm | 1350  ppm | 1800  ppm |
| 0 | 0.094 | 0.094 | 0.094 | 0.094 | 0.094 | 0 | 0.204 | 0.204 | 0.204 | 0.204 | 0.204 |
| 1 | 0.127 | 0.100 | 0.101 | 0.105 | 0.089 | 1 | 0.238 | 0.174 | 0.168 | 0.170 | 0.171 |
| 2 | 0.211 | 0.090 | 0.092 | 0.077 | 0.078 | 2 | 0.385 | 0.159 | 0.093 | 0.165 | 0.157 |
| 3 | 0.361 | 0.108 | 0.086 | 0.090 | 0.079 | 3 | 0.667 | 0.146 | 0.145 | 0.146 | 0.153 |
| 4 | 0.445 | 0.066 | 0.067 | 0.066 | 0.069 | 4 | 1.147 | 0.148 | 0.155 | 0.126 | 0.164 |
| 5 | 0.675 | 0.069 | 0.071 | 0.081 | 0.079 | 5 | 1.299 | 0.161 | 0.142 | 0.142 | 0.144 |
| 6 | 0.680 | 0.063 | 0.071 | 0.076 | 0.073 | 6 | 1.577 | 0.150 | 0.128 | 0.149 | 0.129 |
| OD600 L. rhamnosus in Sodium Bisulfite 9/19/2016 | | | | | | OD600 S. thermophilus in Sodium Bisulfite 10/27/2016 | | | | | |
| Time/  Hours | NP | 900  ppm | 1170  ppm | 1350  ppm | 1800  ppm | Time/  Hours | NP | 900  ppm | 1170  ppm | 1350  ppm | 1800  ppm |
| 0 | 0.079 | 0.079 | 0.079 | 0.079 | 0.079 | 0 | 0.044 | 0.044 | 0.044 | 0.044 | 0.044 |
| 1 | 0.108 | 0.087 | 0.088 | 0.079 | 0.095 | 1 | 0.081 | 0.050 | 0.045 | 0.039 | 0.042 |
| 2 | 0.186 | 0.082 | 0.085 | 0.088 | 0.093 | 2 | 0.149 | 0.069 | 0.036 | 0.042 | 0.029 |
| 3 | 0.319 | 0.087 | 0.072 | 0.072 | 0.079 | 3 | 0.405 | 0.055 | 0.034 | 0.032 | 0.032 |
| 4 | 0.474 | 0.072 | 0.089 | 0.071 | 0.073 | 4 | 0.584 | 0.053 | 0.030 | 0.032 | 0.026 |
| 5 | 0.708 | 0.080 | 0.098 | 0.084 | 0.095 | 5 | 0.821 | 0.063 | 0.044 | 0.041 | 0.038 |
| 6 | 0.941 | 0.074 | 0.079 | 0.073 | 0.090 | 6 | 0.893 | 0.045 | 0.042 | 0.045 | 0.038 |

Table S1E: Data set describing OD600 in NaHSO_3_.

**Table S1F: Colony Forming Units after exposure to Sodium Bisulfite (NaHSO₃)**

| CFU/mL L. casei in Sodium Bisulfite 1/31/2017 | | | | | | CFU/mL L. plantarum in Sodium Bisulfite 1/31/2017 | | | | | |
| --- | --- | --- | --- | --- | --- | --- | --- | --- | --- | --- | --- |
| Time/  Hours | NP | 900  ppm | 1170  ppm | 1350  ppm | 1800  ppm | Time/  Hours | NP | 900  ppm | 1170  ppm | 1350  ppm | 1800  ppm |
| 0 | 4.6E7 | 4.6E7 | 4.6E7 | 4.6E7 | 4.6E7 | 0 | 2.6E8 | 2.6E8 | 2.6E8 | 2.6E8 | 2.6E8 |
| 2 |  | 0 | 0 | 0 | 0 | 2 |  | 5E4^a^ | 5E4^a^ | 5E4^a^ | 5E4^a^ |
| 4 |  | 0 | 0 | 0 | 0 | 4 |  | 0 | 0 | 0 | 0 |
| 6 | 4.6E9 | 0 | 0 | 0 | 0 | 6 | 5.4E9 | 0 | 0 | 0 | 0 |
| CFU/mL L.rhamnosus in Sodium Bisulfite 9/19/2016 | | | | | | CFU/mL S. thermophilus in Sodium Bisulfite 10/27/16 | | | | | |
| Time/  Hours | NP | 900  ppm | 1170  ppm | 1350  ppm | 1800  ppm | Time/  Hours | NP | 900  ppm | 1170  ppm | 1350  ppm | 1800  ppm |
| 0 | 7E6 | 7E6 | 7E6 | 7E6 | 7E6 | 0 | 8.2E7 | 8.2E7 | 8.2E7 | 8.2E7 | 8.2E7 |
| 2 | 5.5E7 | 0 | 0 | 0 | 0 | 2 |  | 6.2E6 | 2.8E7 | 3.2E7 | 1.3E7 |
| 4 | 1.7E8 | 0 | 0 | 0 | 0 | 4 |  | ND | 1E5^a^ | 1E5^a^ | 2E4 |
| 6 | 3E8 | 0 | 0 | 0 | 0 | 6 | 1E10 | 1E5^a^ | 1E5^a^ | 1E5^a^ | 0 |

Table S1F: Data set describing CFU/mL readings in NaHSO_3_. Results are estimated based on 3-8 replicates from drop plates. Numbers with superscript “a” are based on estimates at dilutions of 10^-2^ replicates that had too many colonies to accurately count. ND refers to a reading that was not done due to human error. Zeros “0” represent no growth observed within 24 hours after inoculation. Data used to determine the results of bactericidal studies summarized in Table 3 in manuscript (along with data from Table S1D)
